# Supplementary figures and images for: The impact of Docker containers on the performance of genomic pipelines
Source: PeerJ. 2015 Sep 24;3:e1273. doi: 10.7717/peerj.1273 (PMC4586803; doi:10.7717/peerj.1273)

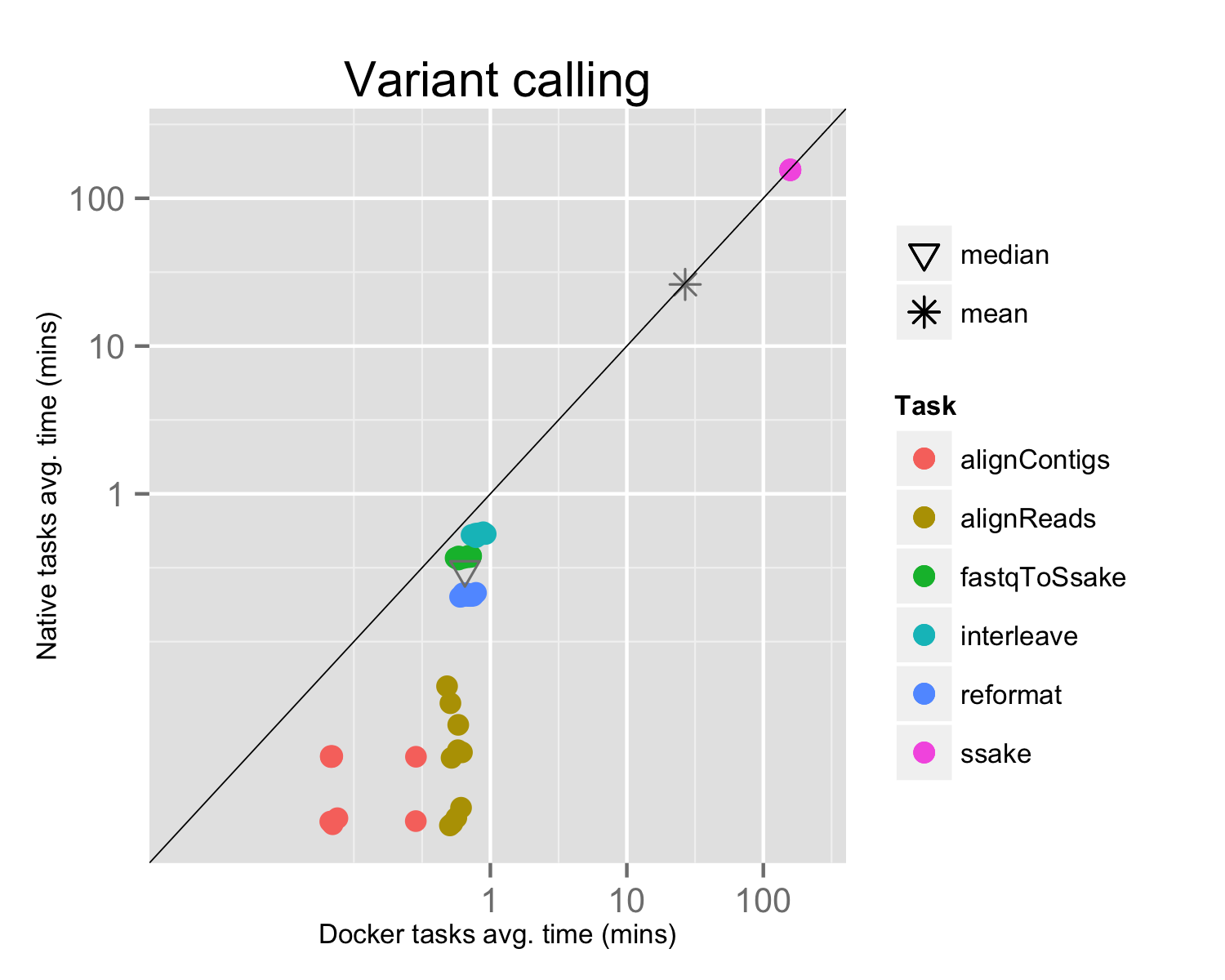

Supplement: Supplemental Information 1 [file peerj-03-1273-s001.zip › docker-benchmarks-peerj5515/nmdp-flow/fig_correlation.png]
