# Supplementary material for: The impact of Docker containers on the performance of genomic pipelines
Source: PeerJ. 2015 Sep 24;3:e1273. doi: 10.7717/peerj.1273 (PMC4586803; doi:10.7717/peerj.1273)
Supplement: Supplemental Information 1 [file peerj-03-1273-s001.zip › docker-benchmarks-peerj5515/piper-nf/docker_8/timeline.html]

### Processes execution timeline

Launch time:    
Elapsed time:

Created with Nextflow -- http://nextflow.io
